# Experimental Investigation on Strength and Stiffness Properties of Laminated Veneer Lumber (LVL)

**DOI:** 10.3390/ma16227194

**Published:** 2023-11-16

**Authors:** Alfredo Romero, Christoph Odenbreit

**Affiliations:** Faculty of Science Technology and Medicine (FSTM), University of Luxembourg, L-4365 Esch-sur-Alzette, Luxembourg; christoph.odenbreit@uni.lu

**Keywords:** timber properties, laminated veneer lumber, LVL, LVL-C, spruce wood, mechanical properties, strength, stiffness

## Abstract

This study presents a testing campaign aimed at evaluating the strength and stiffness properties of laminated veneer lumber (LVL) specimens. LVL is an engineered wood product composed of thin glued wood veneers whose use in construction for structural applications has increased due to its sustainability and enhanced mechanical performance. Despite LVL’s growing popularity, there is a lack of comprehensive information regarding stress–strain responses, failure modes, and the full set of strength and stiffness properties. These are particularly essential when LVL is employed in pure timber structures or composite systems such as steel–timber or timber–concrete load-bearing elements. This research aims to bridge this knowledge gap, focusing on crossbanded LVL panels, known as LVL-C, crafted from Scandinavian spruce wood, which is an LVL product with 20% of crossbanded veneers. The study explores LVL-C mechanical behavior in three primary orthogonal directions: longitudinal, tangential, and radial. A series of mechanical tests, including compression, tension, shear, and bending, was conducted to provide a thorough assessment of the material’s performance. In compression tests, different behaviors were observed in the three directions, with the longitudinal direction exhibiting the highest stiffness and strength. Tensile tests revealed unique stress–strain responses in each direction, with gradual tension failures. Shear tests showcased varying shear stress–strain patterns and failure modes, while bending tests exhibited significant strength and stiffness values in flatwise bending parallel to the grain and flatwise bending perpendicular to the grain. This paper summarizes the comprehensive testing results and discusses the obtained strength and stiffness properties of LVL-C panels, providing valuable insights into their mechanical behavior for engineering applications.

## 1. Introduction

Laminated veneer lumber (LVL) is an engineered wood product that has attracted considerable interest for its ability to harness the inherent strength and durability of wood while addressing some of the constraints related to solid timber size and natural defects. Additionally, LVL manufacturing efficiently makes use of small-diameter logs and lower-grade timber that might otherwise go to waste. LVL products are generally categorized into two main groups based on their layup: (i) those in which veneer layers are oriented with their grain parallel to each other, referred to as LVL-P, and (ii) those in which roughly 20% of the veneer layers have their grain oriented perpendicular to the rest of the veneer layers, known as LVL-C.

As the construction industry faces growing scrutiny for its environmental impact [1], engineered wood products such as CLT and LVL have emerged as a sustainable alternative to conventional materials like concrete and steel [2,3,4,5]. LVL is engineered to deliver exceptional strength and stiffness. Its composite structure, comprising 3–4 mm thick veneer layers that are pressed and bonded with phenolic adhesive, mitigates many of the natural defects that are inherent in solid wood [6]. This leads to a highly uniform material with reduced property variations, setting it apart from traditional timber products. The appeal of LVL extends to its suitability for a wide range of structural applications, from beams and columns to slabs and shear walls. Moreover, investigations have been carried out to implement its use as slabs in conjunction with steel structural beams as steel–timber composite (STC) beams [7,8,9].

There are some investigations on the mechanical properties and behavior of LVL; however, none of them deal with LVL-C made from Scandinavian spruce wood. Chybinski and Polus [10] conducted compression, tension, and bending tests of LVL-P (LVL with the grain of all veneers oriented in the same direction) panels made of spruce and Scots pine; moreover, they built 2D and 3D finite element models and implemented the properties obtained in their tests, showing good agreement with the experimental observations. A comprehensive investigation on the strength and stiffness properties of Radiata pine LVL was presented by Van Beerschoten [11]. Ardalany et al. [12,13] studied the fracture energy, toughness, and strength in tension perpendicular to the grain of LVL crafted from Radiata pine. Similarly, Franke and Quenneville [14] analyzed experimentally the fracture behavior of Radiata pine LVL.

Other studies related to LVL focused on the assessment of LVL with certain variations in its standard structure and/or layup and its response under certain conditions. For example, Bal [15] determined some physical and mechanical properties of LVL reinforced with woven fibers. Sokolovic et al. [16] assessed the bending strength of flexural properties of LVL reinforced with woven carbon fibers. Bakalarz [17] studied the bending response of LVL beams reinforced with carbon-fiber-reinforced polymer (CFRP).

While there are studies that have focused on the mechanical characterization of certain LVL products and on the enhancement of their mechanical response, it is noteworthy that none of them have specifically assessed the strength and stiffness properties of crossbanded LVL derived from Scandinavian spruce wood, as far as the authors of this contribution are aware.

Despite LVL’s increasing use in the construction industry, a comprehensive understanding of its mechanical behavior, including testing procedures, stress–strain responses, and failure modes, remains crucial. The technical literature lacks comprehensive insights into these aspects. The mechanical properties of this engineered timber product (e.g., strength, stiffness, stress–strain response, fracture behavior) are fundamentally important when it is used as a load-bearing structural element in pure timber, steel–timber, or timber–concrete structures. These properties are used to determine the bearing capacity of structural members, their load–deformation behavior, and potential failure modes. Additionally, the properties are essential for developing numerical models of LVL members and structures (e.g., finite element modeling). Hence, to address this existing gap of knowledge concerning the mechanical characterization of LVL-C made of Scandinavian spruce wood, this research focuses on panels made from this wood species.

This investigation studies the LVL-C in its three primary orthogonal directions: longitudinal, tangential, and radial. A series of mechanical tests of LVL-C specimens in compression, tension, shear, and bending has been conducted to comprehensively evaluate the material’s performance in the three orthogonal directions. This contribution provides valuable insights concerning the testing procedures of the material along with values of strength, moduli of elasticity, and shear moduli. In addition, the stress–strain responses and load–deformation response in bending are presented.

This article is organized as follows: a description of the tested material is presented in Section 2.1, the overview of the tests is included in Section 2.2, the testing procedures, specimen characteristics, and test setups are described in Section 3, the results and the respective discussion is provided in Section 4, and, finally, the conclusions are presented in Section 5.

## 2. Material and Tests Overview

### 2.1. Material

Laminated veneer lumber is an engineered wood product made by gluing and layering wood veneers with a thickness of 3–4 mm to produce wood panels with various dimensions and layups. The maximum width (measured in the tangential direction) that can be produced is 2500 mm, and the maximum lengths range from 18 to 25 m depending on the production line. There are two standard types of layups: (i) a layup in which the veneer layers are oriented with their grain running parallel to each other, known as LVL-P; and (ii) a layup in which approximately 20% of the layers of veneer have their grain running perpendicular to the grain of the other veneers, which is known as LVL-C.

**Figure 1:**
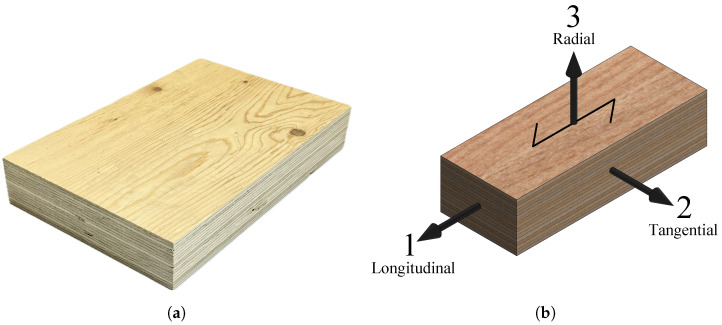
LVL tested in this study: (**a**) picture of the material, and (**b**) directions considered.

For this testing campaign, the specimens were produced from LVL-C panels with a thickness of 51 mm made of Scandinavian spruce wood (i.e., Picea abies) (see Figure 1a). This product is commercialized by Metsä Wood under the brand name of Kerto-Q. It has 17 veneers in total, each veneer with a thickness of 3 mm, where 3 of them are cross veneers distributed within the matrix of the section; hence, the layup is as follows: II-IIIII-IIIII-II, in which ‘I’ represents the veneers whose grain aligns with the longitudinal direction of the timber element and ‘-’ represents the cross veneers.

Due to its nature, timber is anisotropic; however, for engineering purposes, it is considered as an orthotropic material. The three orthogonal directions are linked to the growth directions of the trees, longitudinally and transversely. The strongest and the stiffest direction is often referred to as the longitudinal direction, or grain direction, and it follows the longitudinal growth of the tree. The other two directions are the radial and the tangential direction, which follow the transversal growth of the tree. Figure 1b shows a picture of the LVL material and the definitions of the directions considered in this study for the LVL panels: the longitudinal (L), tangential (T), and radial (R) directions are defined as directions 1, 2, and 3, respectively.

### 2.2. Tests Overview

**Figure 2:**
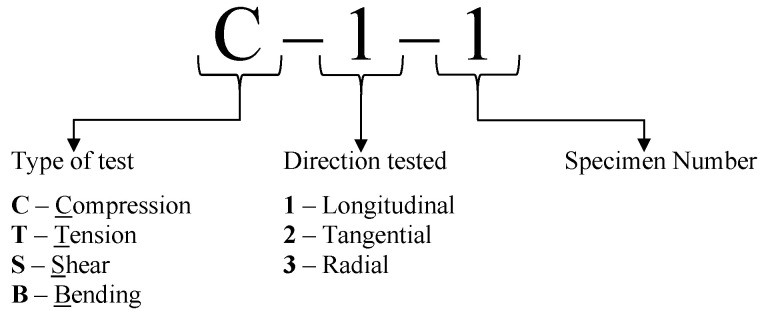
Nomenclature definition for test types and specimens identification.

**Figure 3:**
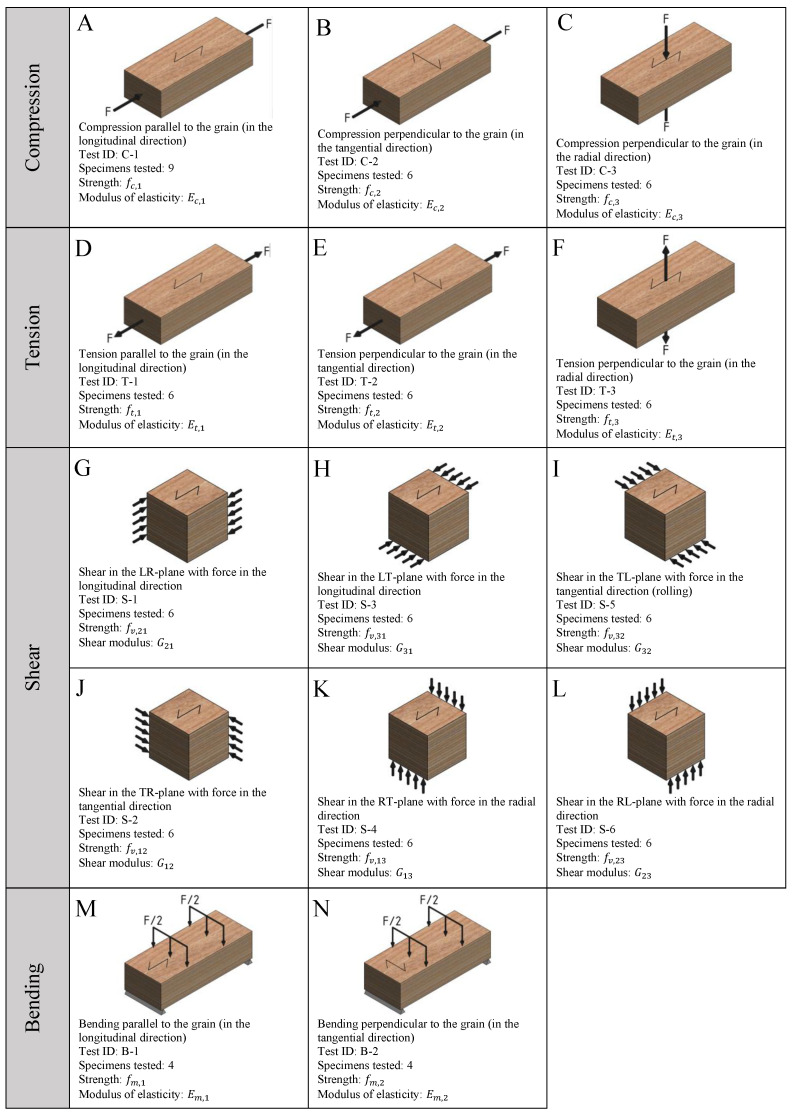
Overview of the tests carried out in this experimental campaign.

The testing campaign includes compression (C-), tension (T-), shear (S-), and bending (B-) tests to determine the stiffness and strength properties of crossbanded LVL in the three main orthogonal directions. A nomenclature was defined to label the specimens and assign IDs to the different tests; this nomenclature (see Figure 2) consists of one letter followed by two digits separated by dashes, where the letter refers to the type of test, the first digit, which is placed after the letter, refers to the direction, and the last digit refers to the specimen number within the sample. Following this nomenclature, an overview of the tests is presented in Figure 3. This figure includes the schematic representations of the specimens grain direction, the test IDs, the direction of the applied loads, and the number of specimens tested in this experimental campaign.

## 3. Methods

### 3.1. General

The tests were conducted in accordance with the standards EN 408 [18] and EN 789 [19], and the strength and stiffness values were estimated following the procedures established in these standards; further details of the calculation procedures can be found in Appendix A. The coefficient of variation and the 5-percentiles values were calculated according to EN 14358 [20].

All the tests were carried out at room temperature conditions. The moisture content of the LVL specimens was measured using a capacitive moisture sensor from ALEMO (FHA 696 MF). The air humidity and temperature were also measured using a digital sensor from ALEMO (FHAD46-Cx) (Sempeter pri Gorici, Slovenia). The average measurements recorded were as follows: moisture content at 12%, air humidity at 46%, and room temperature at 23 °C.

In accordance with standards EN 408 [18] and EN 789 [19], the loading procedures were as follows: (i) a monotonic load was applied to all specimens in displacement-controlled mode, and (ii) specific loading rates were defined for each test to ensure that specimen failure occurred within a specific time frame of 300 ± 120 s.

For the tension and shear tests, custom apparatus were designed. For tension tests, the custom apparatus consist of clamping steel plates in which bolts were used to apply pressure on the specimen; in addition, glue was applied to prevent slip, which could cause drops in forces during the tests. For shear tests, the custom apparatus consist of (i) a set of bearings that allow us to introduce the loading to the specimen with an inclination of 14° with respect to the vertical, such that shear failure is induced through the specimen, and (ii) steel plates as recommended in EN 408 [18]; these plates were glued to the specimen in two parallel faces. Further details of these devices have been included in the respective Section 3.3 Tension Tests and Section 3.4 Shear Tests.

Preliminary tests were conducted to validate the performance of the devices and to define a suitable glue for the tension and shear tests. Two types of glue were tested: (i) two-part epoxy glue (Würth ESK-50), and (ii) methylmethacrylate glue (Würth MAK 38). It was determined that the two-part epoxy glue exhibited superior resistance at the glued interface and a shorter curing time. Consequently, this glue was selected for use in both tensile and shear tests to effectively bond steel and LVL surfaces.

### 3.2. Compression Tests

**Figure 4:**
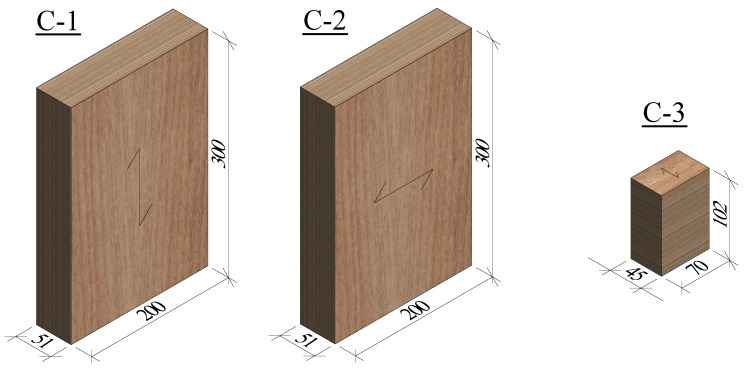
Dimensions in mm of the specimens tested in compression.

The compression tests were performed in the longitudinal (C-1), tangential (C-2), and radial (C-3) directions. The specimens’ dimensions are shown in Figure 4. In the C-1 and C-2 tests, a small preload of 5 kN and 2 kN, respectively, was applied to the specimens; then, the load was applied at a rate of 0.6 mm/min and 1.5 mm/min, respectively. In the C-3 tests, no preload was applied, and the loading rate was set to 4 mm/min.

Nine specimens were tested in C-1 tests, and six specimens in C-2 and C-3 tests. In the C-1 tests, initially, six specimens were tested and these tests were stopped at a load drop of 70 kN. However, after analyzing the results of these initial tests, it was decided to test three additional specimens up to the point of fracture to obtain more detailed information about fracture and the softening branch of the stress–strain curve in the longitudinal direction.

**Figure 5:**
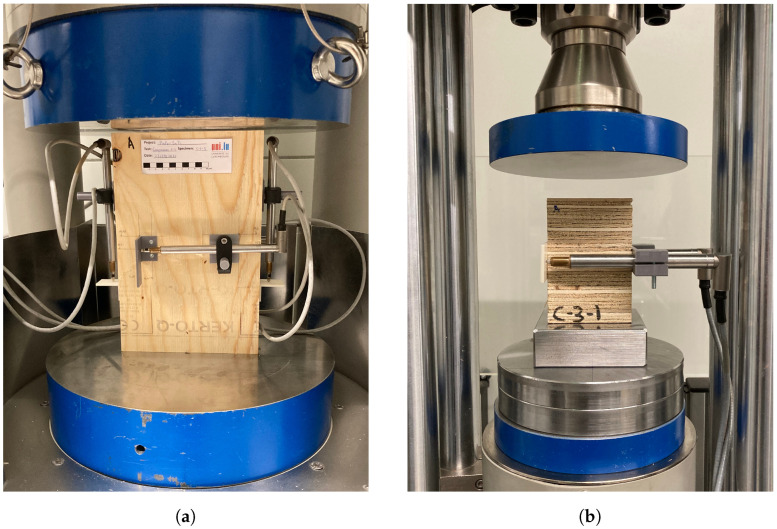
Test setups of: (**a**) compression tests C-1 and C-2, and (**b**) tests C-3.

The test setup of the compression tests is shown in Figure 5. The C-1 and C-2 tests were carried out in a compression testing machine with a capacity of 4 MN and the C-3 tests were carried out in a compression testing machine with a capacity of 300 kN, both machines from TESTING Bluhm & Feuerherdt GmbH (Berlin, Germany).

### 3.3. Tension Tests

**Figure 6:**
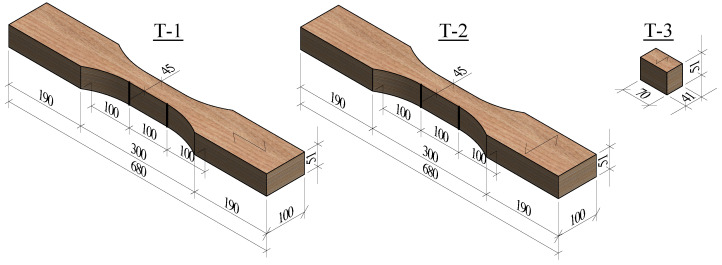
Dimensions in mm of the specimens tested in tension.

The tension tests were performed in the three orthogonal directions: longitudinal (T-1), tangential (T-2), and radial (T-3). Six identical specimens were tested for each direction. For tests T-1 and T-2, the specimens had a coupon shape to induce failure at the central part of the specimen and to measure the local displacements within a well-defined gauge length. The T-3 tests were performed on rectangular prismatic specimens. The shapes and dimensions of the specimens tested in tension are shown in Figure 6. The tests were conducted in a universal machine for compression and tension tests Form+Test UP 500 with a capacity of 100 kN.

**Figure 7:**
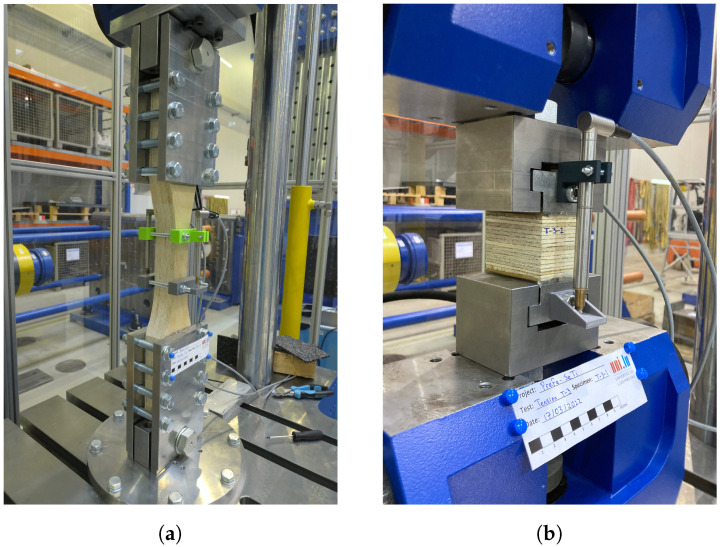
Test setups of: (**a**) tension tests T-1 and T-2, and (**b**) tests T-3.

Custom apparatus were designed and produced to carry out these tests. The custom devices for the T-1 and T-2 tests consist of steel plates with a thickness of 20 mm with drilled holes to allow for the installation of bolts in order to apply pressure and clamp the coupon-shaped specimen from its tabs. To prevent slip and force drops due to slip of the specimen, a two-part epoxy glue was applied at the steel–timber interface of the custom griping plates. In the T-3 tests, the load was transferred to the specimen through custom grips and steel blocks that were fixed to the specimen with a two-part epoxy glue. Images of the test setup of the tension tests are shown in Figure 7.

The load was applied at rates of 0.1 mm/min, 0.3 mm/min, and 0.2 mm/min, respectively, for tests T-1, T-2, and T-3. Local displacements were measured with two LVDT sensors placed at parallel faces of the specimens. In the T-1 and T-2 tests, local displacements were measured in the gage of the specimen within a length of 100 mm. In the T-3 tests, the displacements were measured through the whole length of the specimen (i.e., 51 mm).

### 3.4. Shear Tests

**Figure 8:**
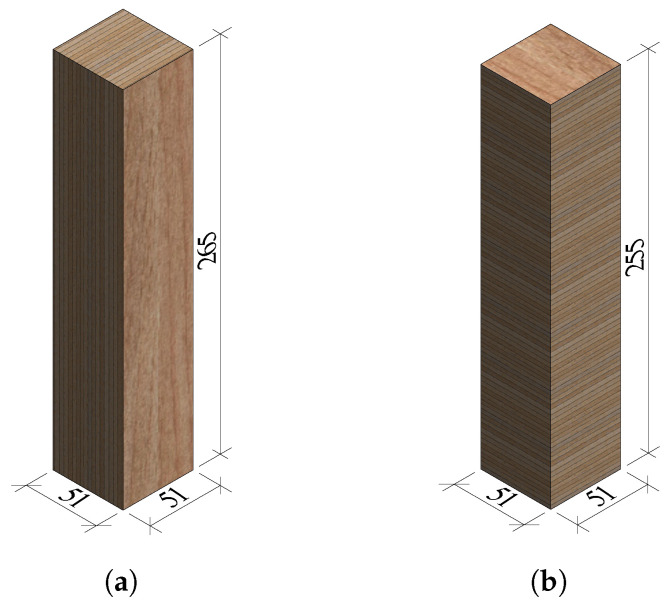
Dimensions in mm of the shear test specimens: (**a**) S-1, S-2, S-3, S-5 and (**b**) S-4, S-6.

Six different shear tests (i.e., S-1, S-2, S-3, S-4, S-5, S-6) were performed in the direction indicated in Figure 3. Six identical specimens were tested for each one of the directions; the dimensions of the specimens are shown in Figure 8. The specimens for tests S-4 and S-6 were formed by stacking and gluing 5 cubic pieces of the timber panels with dimensions 51 × 51 × 51 mm. The tests were conducted in a universal machine for compression and tension tests Form+Test UP 500 with a capacity of 100 kN.

**Figure 9:**
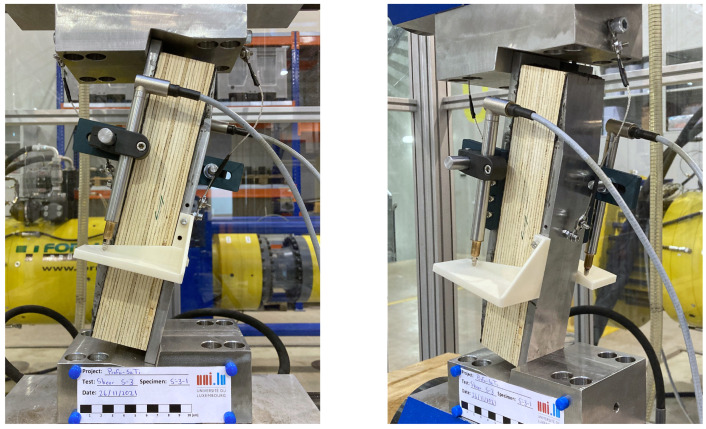
Shear tests setup.

Custom devices were designed and produced for these tests. The top and bottom bearings have a channel that allows us to place and remove a specimen at an inclination of 14° with respect to the vertical line of the applied load. Two steel plates (275 × 51 × 10 mm) with the shape recommended in EN 408 [18] were glued with two-part epoxy glue to the specimen in parallel faces of the specimen in the corresponding shear plane for each test. The differential displacement between the steel plates was measured with two LVDT sensors installed at parallel faces. The test setup is shown in Figure 9.

The loading rate for the S-1 and S-4 tests was set to 0.2 mm/min; for tests S-2 and S-3, it was set to 0.4 mm/min; for tests S-5, it was 0.5 mm/min; and for tests S-6, it was set to 0.6 mm/min.

### 3.5. Bending Tests

**Figure 10:**
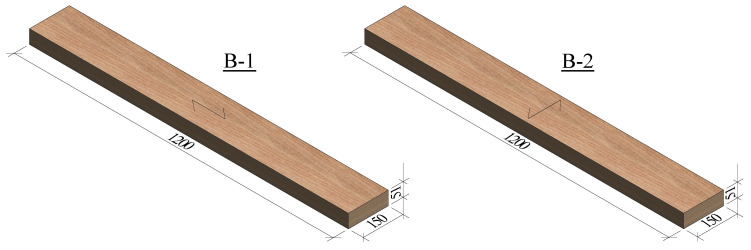
Dimensions in mm of the specimens tested in 4-point bending.

**Figure 11:**
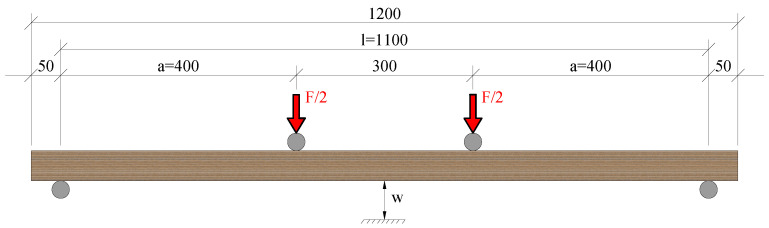
Side view of the position of the loading points and the supports of the bending tests (dimensions given in mm).

**Figure 12:**
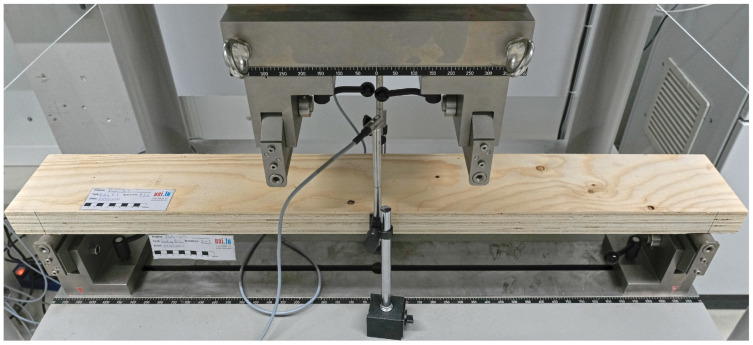
Test setup of the bending tests.

Simply supported panels were tested in 4-point bending. Two different bending tests were executed: flatwise bending parallel to the grain (B-1), and flatwise bending perpendicular to the grain (B-2). Four identical specimens with dimensions 1200 × 150 × 51 mm (see Figure 10) were tested for each type of test. The load was applied at rates of 4.2 mm/min and 4.8 mm/min for tests B-1 and B-2, respectively. The deflection of the panels was measured at midspan at both sides of the panels. The tests were carried out in a machine for bending tests with a capacity of 300 kN from TESTING Bluhm & Feuerherdt GmbH. The positions of the loading points and the supports are depicted in Figure 11 and the test setup is illustrated in Figure 12.

## 4. Results and Discussion

### 4.1. Compression Tests

**Figure 13:**
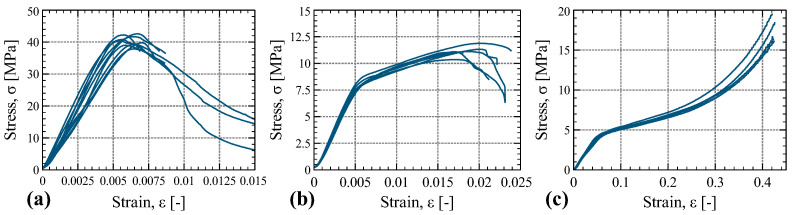
Stress–strain plots of the compression tests C-1 (**a**), C-2 (**b**), and C-3 (**c**).

**Figure 14:**
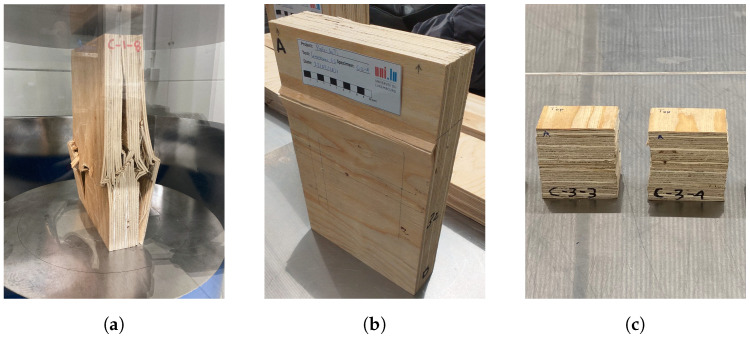
Pictures of specimens tested in compression: C-1 (**a**), C-2 (**b**), and C-3 (**c**).

The stress–strain curves of the compression tests in the three main orthogonal directions are depicted in Figure 13 and some of the tested specimens are illustrated in Figure 14. The response of the material in the longitudinal direction (tests C-1) was characterized by an initial linear monotonic increasing stress–strain relationship, followed by a non-linear response, and, after the peak, a softening branch developed. Out of the nine specimens that were tested in C-1 tests, only three were brought to rupture. After reaching the peak load, crushing of the fibers at the mid-height of the specimen, followed by delamination and opening of the veneers, was observed. In some specimens, shear failure was observed; these specimens showed an inclined crack of crushed veneers through their thickness, with an inclination of about 45°.

The specimens tested in the tangential direction (tests C-2) also exhibited an initial linear behavior followed by a hardening branch and softening after the peak. The tests were stopped before reaching the rupture. In the radial direction (tests C-3), the tested specimens showed an initial linear behavior followed by a hardening region of increasing stress with strain. In this direction, LVL exhibited a large deformation capacity in compression.

### 4.2. Tension Tests

**Figure 15:**
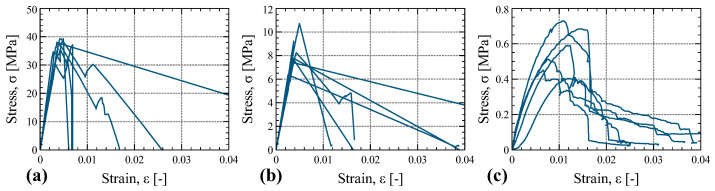
Stress–strain plots of the tension tests T-1 (**a**), T-2 (**b**), and T-3 (**c**).

The stress–strain plots for the tension tests conducted in the three main orthogonal directions are presented in Figure 15. In both the longitudinal (tests T-1) and tangential direction (tests T-2) tensile tests, the stress–strain curves exhibited an initial linear relationship, wherein stresses increased with strain. Subsequently, the specimens reached a maximum force, and failure of the veneers occurred. Following this peak, the stress–strain curve underwent a sharp turn, with stresses decreasing at varying rates across all specimens. Most of the specimens tested in the radial direction (tests T-3) displayed an initial linear stress–strain relationship, characterized by increasing stresses with strain. This was followed by a transition to non-linear behavior, along with a rapid force drop immediately after reaching the peak load, indicating specimen failure.

**Figure 16:**
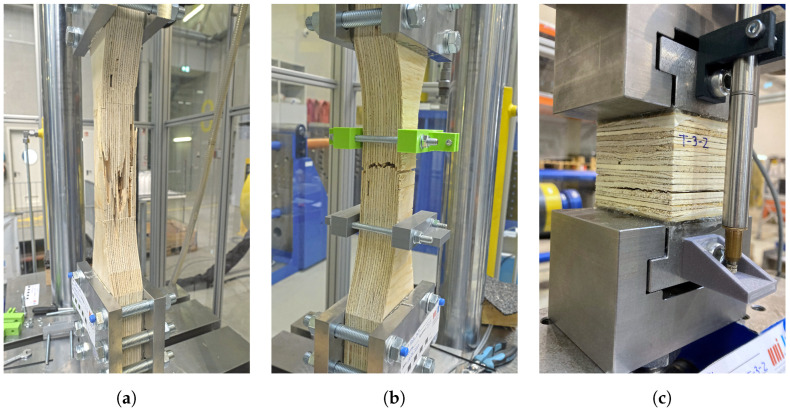
Pictures of specimens tested in tension: T-1 (**a**), T-2 (**b**), and T-3 (**c**).

Figure 16 depicts images of specimens tested in shear at the point of failure. In the case of specimens tested in the longitudinal direction (T-1), the veneers exhibited a gradual tension failure, occurring at various locations without localization to a specific section. Consequently, in some specimens, the post-peak load drop was relatively gradual and, in others, a brief hardening branch emerged after the load drop. In contrast, during the tensile tests conducted in the tangential direction (T-2), in some specimens, the failure was concentrated in a specific section due to the tension failure of the veneers. Furthermore, in the tensile tests conducted in the radial direction (T-3), the observed failure mode was in some cases localized at a well-defined horizontal plane. At this plane, the fibers of a specific wood layer were pulled apart. In other cases, the tensile force induced shear failure through the veneers of the specimen.

### 4.3. Shear Tests

**Figure 17:**
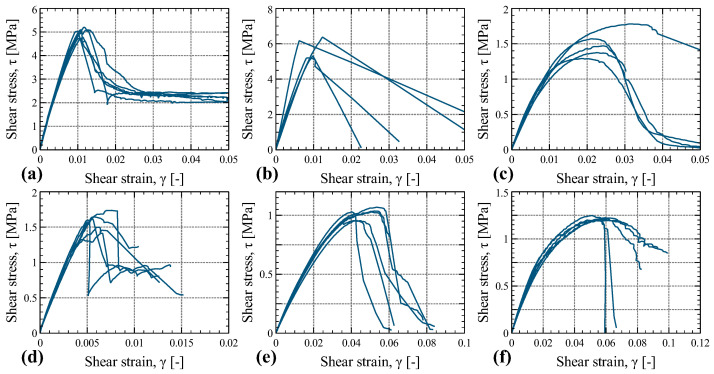
Shear stress vs. shear strain curves of the shear tests: S-1 (**a**), S-2 (**b**), S-3 (**c**), S-4 (**d**), S-5 (**e**), and S-6 (**f**).

The shear stress vs. shear strain plots for the six types of shear tests conducted in this experimental study (refer to Figure 3) are presented in Figure 17. In each of the six defined types of shear tests, six specimens were tested. Nevertheless, in the case of tests S-2, two of the specimens experienced failures at the bonding steel–timber interface. Similarly, in tests S-3, one specimen exhibited glue failure. Consequently, the results of these particular tests were excluded from the calculations and were not considered in the final analysis.

Generally, the stress–strain responses of the tested samples followed a similar pattern. There was an initial linear increase in stress with increasing strain, followed by a subsequent force drop that indicated specimen failure. Notably, in tests S-1 and S-2, following this force drop, a plateau phase was observed in which there was no further increase or decrease in load with increasing strain.

**Figure 18:**
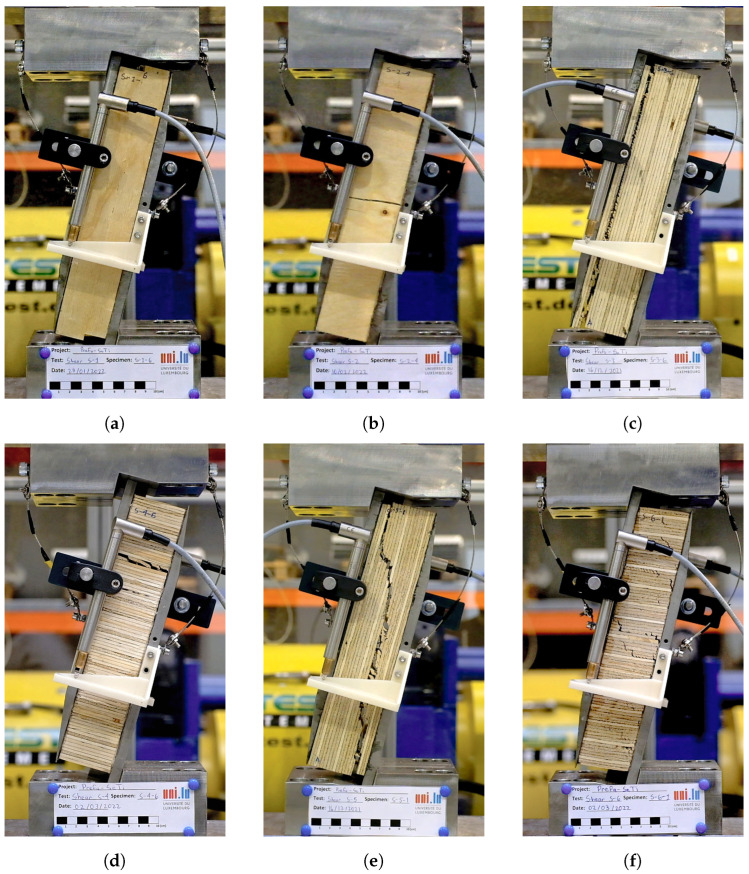
Pictures of specimens tested in shear: S-1 (**a**), S-2 (**b**), S-3 (**c**), S-4 (**d**), S-5 (**e**), and S-6 (**f**).

Figure 18 presents images showcasing typical failure modes of the tested specimens. In the case of tests S-1, the specimens exhibited a vertical shear plane extending from the top to the bottom. However, this plane did not cut straight through the thickness of the specimen, resulting in the specimen remaining in one piece, with friction between the cut veneers preventing separation. In the S-2 tests, the shearing planes were horizontal and followed the grain direction. The failure mode observed in the S-3 tests was localized to a single veneer in the vertical direction of the specimen, with shear failure occurring along that specific wood layer. For specimens in tests S-4, the failure occurred along a cutting plane aligned horizontally with one of the LVL layers. In the case of tests S-5, the failure crack due to the induced shear propagated from one corner on the top of the specimen to the opposite corner on the bottom. Lastly, in tests S-6, cracks appeared throughout the wood veneers of the specimen, resulting in gradual softening and eventual rupture through some of the layers.

### 4.4. Bending Tests

**Figure 19:**
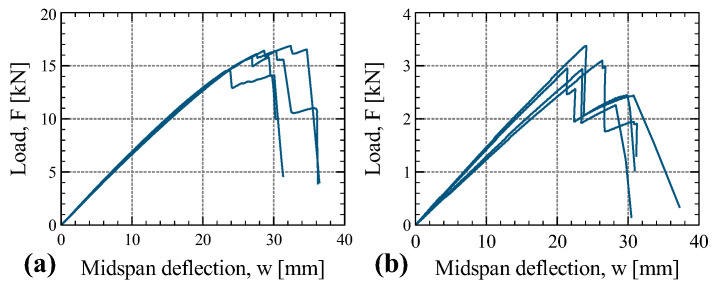
Load–deflection curve of tests B-1 (**a**) and B-2 (**b**).

The load–deflection curves (refer to Figure 19) for two types of bending tests are presented in this investigation: (i) bending flatwise parallel to the grain in the longitudinal direction (B-1 tests), and (ii) bending flatwise perpendicular to the grain in the tangential direction (B-2 tests). The peak loads achieved in these two types of tests differ significantly in magnitude, with B-1 tests reaching peak loads approximately five times greater than those observed in B-2 tests. Nevertheless, the specimens of both test types exhibited a similar load–slip response pattern. Initially, midspan deflection increased linearly up to the point of peak load, followed by a subsequent force drop attributed to the failure of some veneers.

In some specimens, following this initial drop, the panel exhibited the ability to carry additional load, resulting in a short branch where load increased in tandem with midspan deflection. Ultimately, a sudden load drop marked the failure of the specimen.

**Figure 20:**
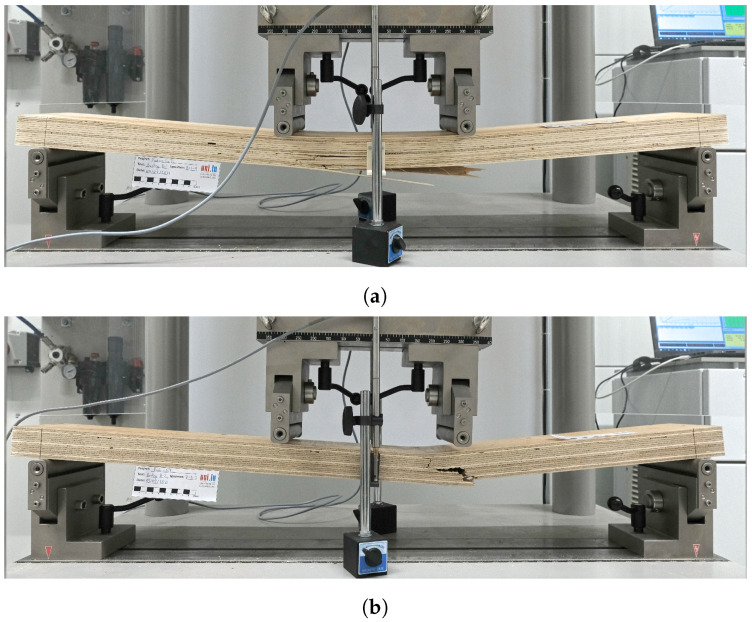
Pictures of specimens tested in bending: B-1 (**a**) and B-2 (**b**).

Images of some of the tested specimens are presented in Figure 20. In the bending tests in the longitudinal direction B-1, gradual failure of the bottom fibers was observed occurring at different locations within the region of the points of load application. A similar failure mode was observed in the bending tests conducted in the tangential direction, where the failure was initiated at the bottom fibers near one of the points of load application.

### 4.5. Summary of Strength and Stiffness Values

A summary of the calculated strength and stiffness properties is presented in Table 1 and Table 2, respectively. The values were computed according to EN 408 [18] and EN 789 [19]; more details of the calculation procedures can be found in Appendix A. The 5th percentile (5th P) values of these properties were estimated according to EN 14358 [20].

The longitudinal direction of the LVL-C tested in this study exhibited the best performance in terms of strength and stiffness for both tension and compression, followed by the tangential direction and the radial direction, which showed a very low stiffness and turned into non-linear plastic behavior at quite small stresses. Similarly, the panels tested in bending performed the best in the flatwise bending tests parallel to the grain, and the bending strength and stiffness of the panels tested in flatwise bending perpendicular to the grain were about one fifth of the values obtained in the bending parallel to the grain.

## 5. Conclusions

This study aimed to fill a significant knowledge gap by providing comprehensive information about the mechanical properties of laminated veneer lumber (LVL-C) made from Scandinavian spruce wood. The investigation included the determination of strength, moduli of elasticity, and shear moduli through a series of tests, including compression, tension, shear, and bending. Additionally, the study presents the testing procedures, load–deformation responses, and descriptions of observed failure modes.

The mechanical properties obtained in this research are crucial for analyzing the load–deformation behavior and capacity of various structural components implementing LVL-C crafted from Scandinavian spruce wood, including pure timber, steel–timber, and timber–concrete load-bearing elements. These properties can be employed in analytical calculations and incorporated into numerical models, such as finite element models, to explore the deformation and failure of structural members.

In addition to the property values, stress–strain responses, and load–deformation behavior, the key findings of this study are as follows:

- In compression and tension tests, the longitudinal direction exhibits the highest strength and stiffness, followed by the tangential and radial directions.
- Stress–strain responses in compression differ among the three directions, with the longitudinal direction showing softening after reaching the peak, the tangential direction exhibiting hardening after the proportional limit, and the radial direction demonstrating significant deformation capacity and a hardening branch after the proportional limit.
- Tension tests shows a sharp transition in which the load starts to decrease, which happens when the fibers in the matrix of the specimen fail in tension; however, the failure in some cases is not sudden as the failure is not localized at a specific section but rather in an irregular pattern, and friction remains within the fractured veneers.
- Shear tests S-3 to S-6 exhibit a near-brittle post-peak response, marked by sudden load drops after reaching the peak. In contrast, shear tests S-1 and S-2 show a post-peak behavior with a gradual load decrease due to ongoing inter-layer friction.
- Bending tests reveal that failure occurs at the soffit. Gradual failure on a layer-by-layer basis is observed, with load drops occurring when the most stressed layers of the soffit fail. Ultimately, a final sudden load drop is associated with the fracture of the matrix.

These findings contribute valuable insights into the mechanical behavior of LVL-C crafted with Scandinavian spruce wood, enhancing its applicability in various engineering contexts.

## Figures and Tables

**Table 1:** Summary of strength properties.

Strength Property	Notation	From Figure 3	Strength [MPa]		
			Mean *	5th P *	CV * [%]
Compression strength parallel to the grain, edgewise (in the longitudinal direction)	$f_{c,1}$	A	40.41	36.11	3.91
Compression strength perpendicular to the grain, edgewise (in the tangential direction)	$f_{c,2}$	B	11.14	9.83	4.44
Compression strength perpendicular to the grain, flatwise (in the radial direction)	$f_{c,3}$	C	3.99	2.99	10.64
Tension strength parallel to the grain, edgewise (in the longitudinal direction)	$f_{t,1}$	D	37.76	34.10	4.11
Tension strength perpendicular to the grain, edgewise (in the tangential direction)	$f_{t,2}$	E	8.33	5.47	17.68
Tension strength perpendicular to the grain, flatwise (in the radial direction)	$f_{t,3}$	F	0.56	0.31	23.04
Shear in the LR-plane with force in the longitudinal direction (edgewise shear parallel to the grain)	$f_{v,21}$	G	4.98	4.40	3.79
Shear in the LT-plane with force in the longitudinal direction (flatwise shear parallel to the grain)	$f_{v,31}$	H	1.50	1.03	12.69
Shear in the TL-plane with force in the tangential direction (flatwise shear perpendicular to the grain)	$f_{v,32}$	I	1.01	0.89	4.57
Shear in the TR-plane with force in the tangential direction (edgewise shear perpendicular to the grain)	$f_{v,12}$	J	5.76	4.16	10.28
Shear in the RT-plane with force in the radial direction (edgewise shear perpendicular to the grain)	$f_{v,13}$	K	1.58	1.32	7.17
Shear in the RL-plane with force in the radial direction (edgewise shear perpendicular to the grain)	$f_{v,23}$	L	1.21	1.07	1.51
Bending parallel to the grain in the longitudinal direction (flatwise bending parallel to the grain)	$f_{m,1}$	M	49.01	40.82	6.16
Bending perpendicular to the grain in the tangential direction (flatwise bending perpendicular to the grain)	$f_{m,2}$	N	9.51	7.83	6.53

* Evaluated according to EN 14358 for the number of specimens tested in each type of test.

**Table 2:** Summary of stiffness properties.

Stiffness Property	Notation	From Figure 3	Stiffness [MPa]		
			Mean *	5th P *	CV * [%]
MoE in compression parallel to the grain, edgewise (in the longitudinal direction)	$E_{c,1}$	A	7 917.08	7 626.51	17.29
MoE in compression perpendicular to the grain, edgewise (in the tangential direction)	$E_{c,2}$	B	1 764.47	1 737.85	4.44
MoE in compression perpendicular to the grain, flatwise (in the radial direction)	$E_{c,3}$	C	95.49	93.83	5.74
MoE in tension parallel to the grain, edgewise (in the longitudinal direction)	$E_{t,1}$	D	10 680.01	10 174.84	15.67
MoE in tension perpendicular to the grain, edgewise (in the tangential direction)	$E_{t,2}$	E	2 199.90	2 140.77	8.91
MoE in tension perpendicular to the grain, flatwise (in the radial direction)	$E_{t,3}$	F	92.05	84.30	27.88
Shear modulus in the LR-plane with force in the longitudinal direction (edgewise shear parallel to the grain)	$G_{v,21}$	G	582.82	573.59	5.25
Shear modulus in the LT-plane with force in the longitudinal direction (flatwise shear parallel to the grain)	$G_{v,31}$	H	123.26	119.48	9.23
Shear modulus in the TL-plane with force in the tangential direction (flatwise shear perpendicular to the grain)	$G_{v,32}$	I	34.27	33.75	4.86
Shear modulus in the TR-plane with force in the tangential direction (edgewise shear perpendicular to the grain)	$G_{v,12}$	J	765.22	691.18	25.87
Shear modulus in the RT-plane with force in the radial direction (edgewise shear perpendicular to the grain)	$G_{v,13}$	K	342.96	336.90	5.86
Shear modulus in the RL-plane with force in the radial direction (edgewise shear perpendicular to the grain)	$G_{v,23}$	L	49.09	44.99	7.90
Global MoE in bending parallel to the grain in the longitudinal direction (flatwise bending parallel to the grain)	$E_{m,1}$	M	10 193.74	10 003.05	1.84
Global MoE in bending perpendicular to the grain in the tangential direction (flatwise bending perpendicular to the grain)	$E_{m,2}$	N	2 004.94	1 944.65	8.04

* Evaluated according to EN 14358 for the number of specimens tested in each type of test.

**Figure A1:**
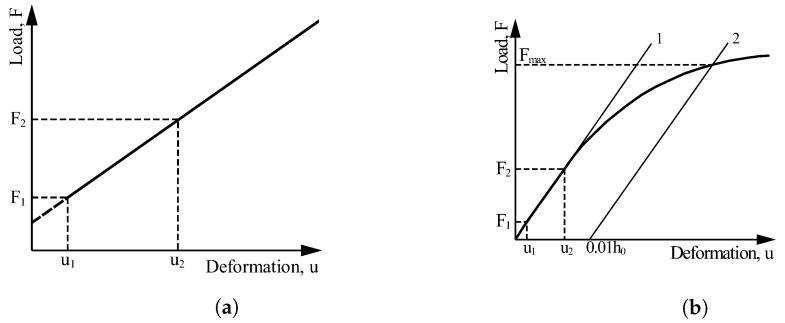
Plots illustrating the parameters of Formulas (A1) and (A2) for (**a**) compression tests (C-1 and C-2), tension tests (T-1, T-2, and T-3), and (**b**) compression tests (C-3).

**Figure A2:**
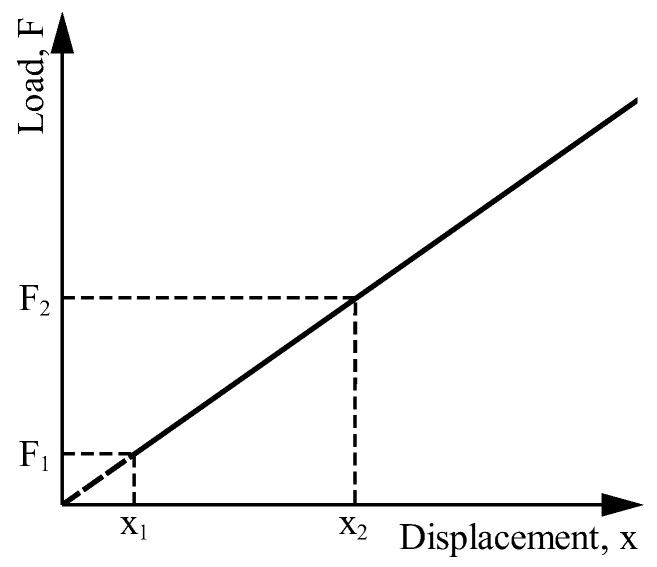
Plot illustrating the parameters of Formulas (A3) and (A4) for shear tests (S-1 to S-6).

**Figure A3:**
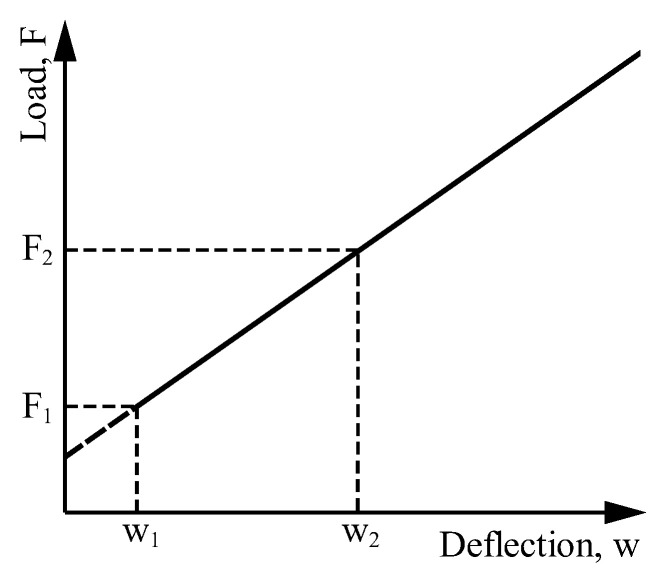
Plot illustrating the parameters of Formulas (A5) and (A6) for bending tests (B-1 and B-2).

## Data Availability

Data are contained within the article.

## Appendix A. Calculation Procedures of Strength and Stiffness Properties

The calculation procedures followed in this study for the computation of the presented strength and stiffness values have been taken from EN 408 [18] and EN 789 [19]. The formulas and the figures presented in this annex were reproduced from these standards.

### Appendix A.1. Compression and Tension Tests

The calculation of the modulus of elasticity (*E*) and the strength (*f*) in compression and tension tests was carried out according to Formulas (A1) and (A2):

(A1) $$E=\frac{(F_{2}-F_{1})l}{(u_{2}-u_{1})A}$$

(A2) $$f=\frac{F_{max}}{A}$$

where:

$F_{2}-F_{1}$ is an increment in load on the regression line with a correlation coefficient of 0.99 or better;

*l* is the initial gauge length;

$u_{2}-u_{1}$ is the increment in deformation corresponding to $F_{2}-F_{1}$;

$F_{max}$ is the maximum force reached during the test;

*A* is the cross-sectional area of the specimen.

The determination of the modulus of elasticity in compression tests (C-1 and C-2) and in tension tests (T-1, T-2, and T-3) was performed by considering a load increment ($F_{2}-F_{1}$) between 10% and 40% of the maximum applied load ($F_{max}$) as illustrated in Figure A1a. In compression in the radial direction (C-3), the load–deformation curves differ from the typical curves obtained in the other tests. In this case, the hardening branch increases monotonically and there is no clear turning point in which a maximum load is reached followed by softening and failure. Therefore, a different iterative procedure is defined for these tests according to EN 408 [18] as follows:

1. Using the test results, plot the load–deformation curve in the form shown in Figure A1b.
2. Calculate $F_{1}$ ($0.1F_{max}$) and $F_{2}$ ($0.4F_{max}$) and determine where these values intersect the load–deformation curve.
3. Through these two points, draw the straight line 1 as shown in Figure A1b.
4. Parallel to line 1, draw line 2 having its origin at load F=0 and at a distance from it equivalent to a deformation of $0.1h_{0}$ as shown in Figure A1b, where $h_{0}$ is the initial height of the specimen.
5. Where line 2 intersects the curve of the test results is $F_{max}$. If the value of $F_{max}$ as determined is within 5% of the initially $F_{max}$ value estimated in step 2, then that value may be used to determine the compressive strength; otherwise, repeat the procedure until a value of $F_{max}$ within that tolerance is obtained.

### Appendix A.2. Shear Tests

The calculation of the shear modulus (*G*) and the shear strength ($f_{v}$) in shear tests was performed according to Formulas (A3) and (A4):

(A3) $$G=\frac{\Delta \tau }{\Delta \gamma }=\frac{(F_{2}-F_{1})\cdot \cos\alpha \cdot t}{l\cdot b\cdot (x_{2}-x_{1})}$$

(A4) $$f_{v}=\frac{F_{max}\cdot \cos\alpha }{l\cdot b}$$

where:

τ is an increment in shear stress;

γ is an increment in shear strain;

$F_{2}-F_{1}$ is an increment in load on the regression line with a correlation coefficient of 0.99 or better;

$x_{2}-x_{1}$ is the increment in relative displacement of the parallel steel plates corresponding to $F_{2}-F_{1}$;

$F_{max}$ is the maximum applied load;

α is the angle between the longitudinal direction of the specimen and the direction of the applied load;

*l* is the length of the specimen;

*b* is the width of the specimen;

*t* is the thickness of the specimen.

The computation of the shear modulus from shear tests (S-1 to S-6) was performed by considering a load increment ($F_{2}-F_{1}$) between 10% and 40% of the maximum applied load ($F_{max}$) as illustrated in Figure A2.

### Appendix A.3. Bending Tests

The calculation of the global bending modulus of elasticity ($E_{m}$) and the bending strength ($f_{m}$) in bending tests was performed according to Formulas (A5) and (A6):

(A5) $$E_{m}=\frac{3al^{2}-4a^{3}}{2bh^{3}\left(2\cdot \frac{w_{2}-w_{1}}{F_{2}-F_{1}}-\frac{6a}{5Gbh}\right)}$$

(A6) $$f_{m}=\frac{3aF_{max}}{bh^{2}}$$

where:

*l* is the distance between the two supports;

*a* is the distance between a loading position and the nearest support;

*b* is the width of the specimen;

*h* is the height of the specimen;

$F_{2}-F_{1}$ is an increment in load on the regression line with a correlation coefficient of 0.99 or better;

$w_{2}-w_{1}$ is the increment in midspan deflection corresponding to $F_{2}-F_{1}$;

*G* is the shear modulus;

$F_{max}$ is the maximum applied load.

The estimation of the global modulus of elasticity in bending was carried out by considering a load increment ($F_{2}-F_{1}$) between 10% and 40% of the maximum applied load ($F_{max}$) as illustrated in Figure A3.
